# Bibliometric review of hotspots and trends of nursing management from 2014 to 2023

**DOI:** 10.1097/MD.0000000000050236

**Published:** 2026-08-14

**Authors:** Yining Wang, Hailin Chu, Ya Xin, Xuyao Liu, Yongxia Ren

**Affiliations:** a Department of Nursing, Tianjin Eye Hospital, Tianjin, China; b Department of Nursing, Tianjin Key Laboratory of Ophthalmology and Visual Science, Tianjin Eye Institute, Tianjin, China; c Department of Nursing, Nankai University Affiliated Eye Hospital, Nankai University, Tianjin, China; d Department of Nursing, Clinical College of Ophthalmology, Tianjin Medical University, Tianjin, China.

**Keywords:** bibliometric, citation analysis, H-index, nursing management

## Abstract

**Background:**

Nursing management is an important part of disease prevention, treatment, and rehabilitation, and it is a focus of nursing research. However, no bibliometric analysis has been conducted on this topic. This study aimed to reveal research trends and provide references for future research directions.

**Methods:**

Relevant publications between 2014 and 2023 were searched from the Web of Science Core Collection on January 9, 2024. Visualizing scientific landscapes viewer, Bibliometrix, and CiteSpace software were used to analyze research trends and hotspots in this field.

**Results:**

A total of 3069 nursing management publications were included in this study. Over the decade, global research on nursing management has developed rapidly. The 3 countries with the highest number of publications were the United States, China, and Australia. Research cooperation between countries and China has been increasing annually. The University of São Paulo in Brazil had the highest number of publications. The top journal contributing to this field was the *Journal of Nursing Management*. The terms “quality of life,” “primary care,” and “nurse manager” were the most frequently used keywords in the field.

**Conclusion:**

Nursing management research is increasingly focusing on patients’ quality of life, psychological status, and primary health care. The findings of this study will be very helpful in understanding future research directions for nursing management and will positively guide the formulation of nursing management policies and the development of the nursing profession.

## 1. Introduction

Since the beginning of the 21st century, with rapid economic and social development, the rates of disease diagnosis and treatment have continuously improved, and patients’ demand for high-quality nursing services has gradually increased.^[1]^ The improvement in nursing quality and standards depends on the progress of nursing management methods and theories.^[2]^ Meanwhile, with the development of medical technology and the continuous improvement in the level of specialization, the demands on nurses’ theoretical knowledge, professional ability, and work efficiency in clinical settings are also increasing.^[3]^ All these factors have led researchers to realize the importance of nursing management research, and the number of research papers on nursing management is increasing annually.^[4]^ Nursing management is the application of scientific theories of management to nursing and is a professional field of management that can make more efficient use of organizational resources, such as human, material, and financial resources, and improve the effectiveness of nursing organizations when nursing managers fully realize the advantages of management functions such as planning, organizing, leading, and controlling.^[5]^ The main goal of hospital work is to strengthen the level of nursing management and improve the quality of nursing staff and patient satisfaction. With the development of information science and technology, especially artificial intelligence (AI) technology, many new changes have taken place in nursing management methods, and many new popular topics and methods have emerged in nursing management research.^[6]^

Bibliometric analysis allows for a comprehensive evaluation of the literature on nursing management by analyzing a large number of publications.^[7]^ This analysis provides an overview of the current state of global research in this field, as well as a prediction of popular research topics and development trends. The purpose of this study was to use the Web of Science Core Collection (WoSCC) database to analyze the nursing management literature and to conduct a bibliometric analysis of the studies associated with relevant publications to quickly understand the historical development, research trends, and most relevant journals in the field. Therefore, we searched all the literature on nursing management from 2014 to 2023 through the WoSCC database and analyzed it with visual analysis software such as Visualizing Scientific Landscapes (VOS) viewer, Bibliometrix, and CiteSpace.

## 2. Materials and methods

Ethical approval was not required for our study, as the data were derived from publicly available sources in the WoSCC, and our study did not involve any experiments on humans or animals.

### 2.1. Data source and search strategy

**Figure 1.**
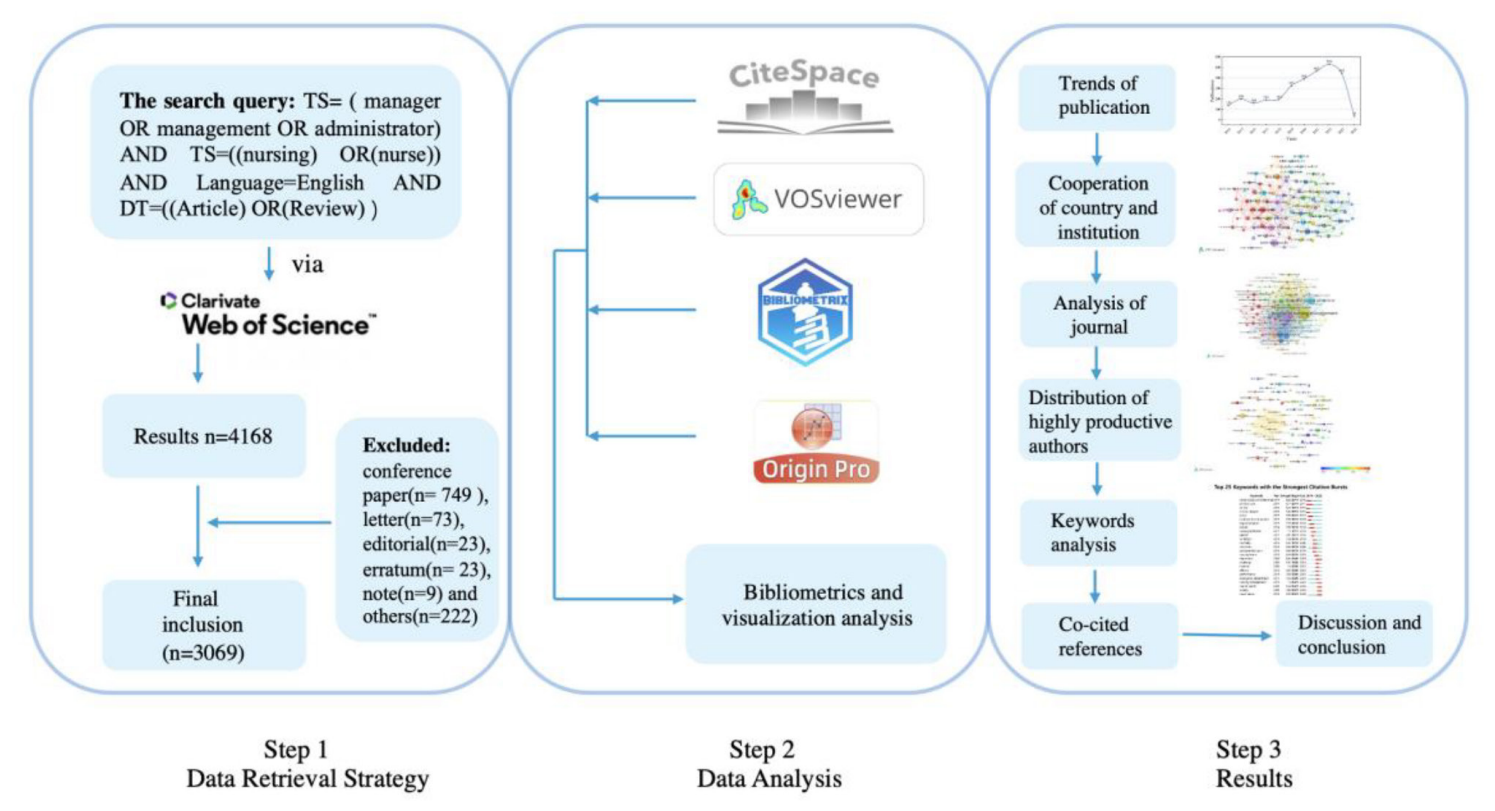
The search strategy and screening procedure. VOS = visualizing scientific landscapes.

The data source used in this study is the WoSCC database, which is widely used in bibliometric research and is a professional database covering most disciplines and containing the largest number of academic studies. The WoSCC database is publicly accessible, and the data are easy to obtain. Accordingly, we conducted a literature search for publications from 2014 to 2023 in the WoSCC database. To avoid bias caused by database updates, we conducted the literature search on January 9, 2024. The following search formula was used: TS = (manager OR management OR administrator OR administration) AND TS = (nursing OR nurse)AND Language = English. A total of 4168 documents of all types were retrieved, including articles (n = 2,793, 67.01%), conference papers (n = 749, 17.97%), reviews (n = 276, 6.62%), letters (n = 73, 1.75%), editorials (n = 23, 0.55%), errata (n = 23, 0.55%), notes (n = 9, 0.22%), and others (n = 222, 5.33%). Two authors (Yining Wang and Hailin Chu) independently retrieved the relevant literature from the database. When the search results of the 2 authors were inconsistent and there was disagreement, they were submitted to a third investigator (Yongxia Ren) for further review. The search strategy and screening procedure are illustrated in Figure 1.

### 2.2. Inclusion and exclusion criteria

Inclusion criteria:

1. Original research articles and review articles.
2. Publications focused on nursing management.
3. Publications in English (for WoSCC).

Exclusion criteria:

1. Conference abstracts, editorials, letters, news items, and retracted publications.
2. Publications where nursing management was not the primary research focus.
3. Duplicate publications.

### 2.3. Data analysis and analysis tools

After carefully reading the titles, abstracts, and keywords of the retrieved studies, we eliminated those with poor relevance to the topic of interest. Ultimately, only 3069 articles and reviews were included in this study. The data for our study were downloaded from the WoSCC in “plain text” format with a “full record and cited references.” Each document included information such as author, country, institution, keywords, publication data, and references.

We used bibliometric analysis software such as VOSviewer (version 1.6.20; Leiden University, the Netherlands), CiteSpace (version 6.4.R1; Drexel University, Philadelphia), R (version 4.5.0; Vienna, Austria) with the Bibliometrix package, and Origin2024 (version 10.3; OriginLab, United States) to analyze and visualize the data of the 3069 documents. VOSviewer is one of the most common software programs used to analyze and visualize bibliometric data on countries, institutions, journals, and authors. CiteSpace is a free application that detects and visualizes trends and patterns in scientific papers through advanced knowledge domain visualization methods. We used CiteSpace to construct the country cooperation map, co-cited literature, keyword time zone map, and literature and keyword burst map, and to perform a visual analysis of the research status, popular topics, and frontiers. We used R (version 4.5.0) with the Bibliometrix package to calculate journal historical changes, generate visual charts, and predict journal publishing trends. Parameters were configured as follows: time slicing from 2014 to 2023 (1 year per slice); term sources selected from Title, Abstract, Author Keywords, and Keywords Plus; node type set as Keyword; selection criteria using the g-index (*k* = 10) for term extraction; and pruning of the merged network using the Pathfinder algorithm. The analysis emphasized transparency and reproducibility. The H-index is another major indicator of academic impact that can be obtained from the WoSCC. The H-index was used to evaluate the scientific impact of a scholar, a country, or an author. It refers to H articles in the literature that have been cited by other scholars at least H times.

## 3. Results

### 3.1. Trend analysis of publications

**Figure 2.**
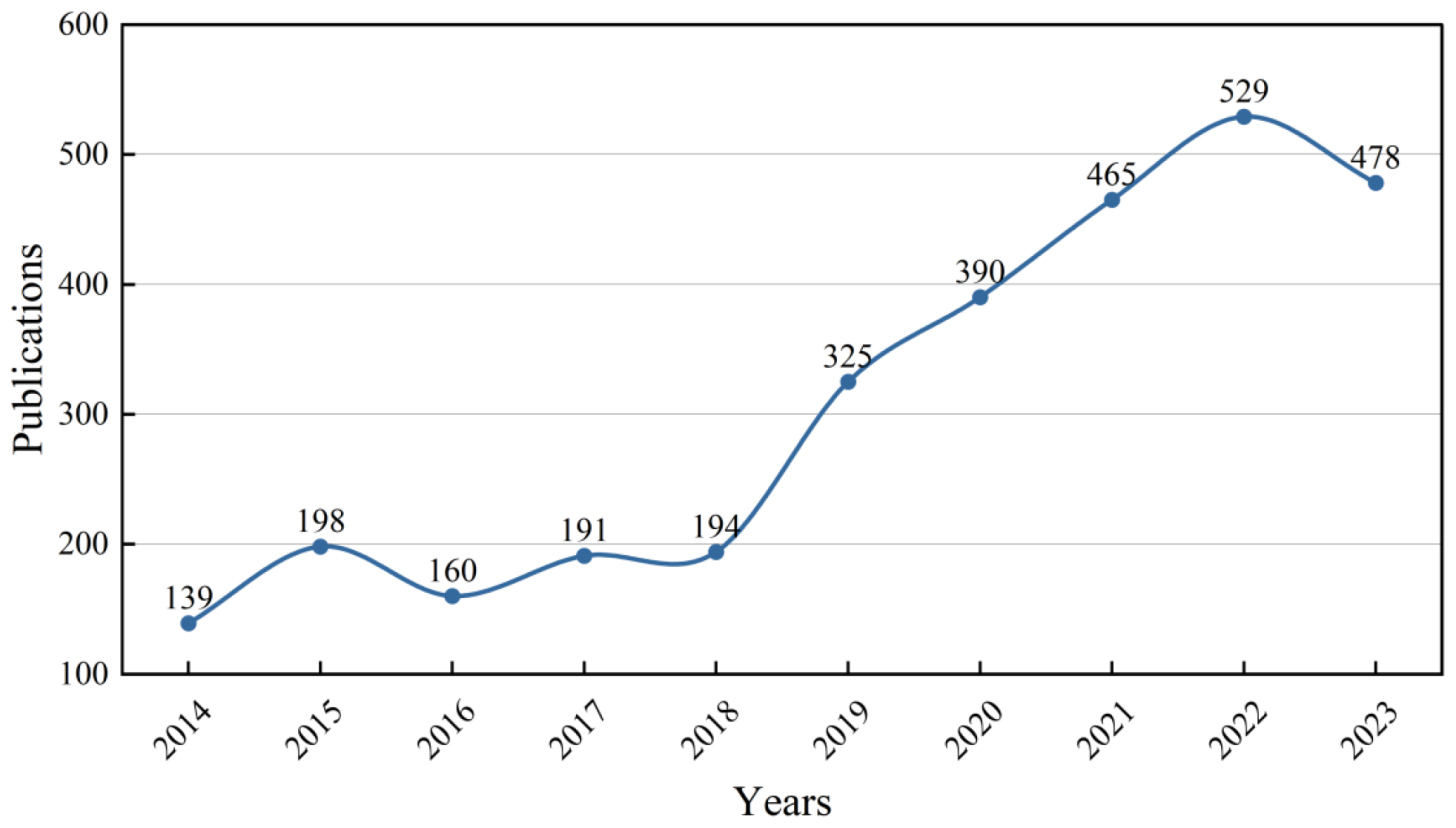
Annual publication trend.

In this study, we retrieved 3069 papers related to nursing management from the WoSCC database that were published in the past 10 years for bibliometric analysis. In 2014, there were only 139 papers related to nursing management in the WoSCC database, and this number increased in 2015; however, the number of published papers slightly declined to 160 in 2016, after which it increased annually, reaching a peak of 529 in 2022. Although the number of papers published in 2023 slightly dropped to 478, this was still more than 3 times that of 2014. Overall, enthusiasm for nursing management research was gradually on the rise. In particular, after 2018, the number of papers increased significantly, which might be related to the application of AI technology to nursing management. Figure 2 shows the number of papers published each year.

### 3.2. Analysis of countries/regions

**Figure 3.**
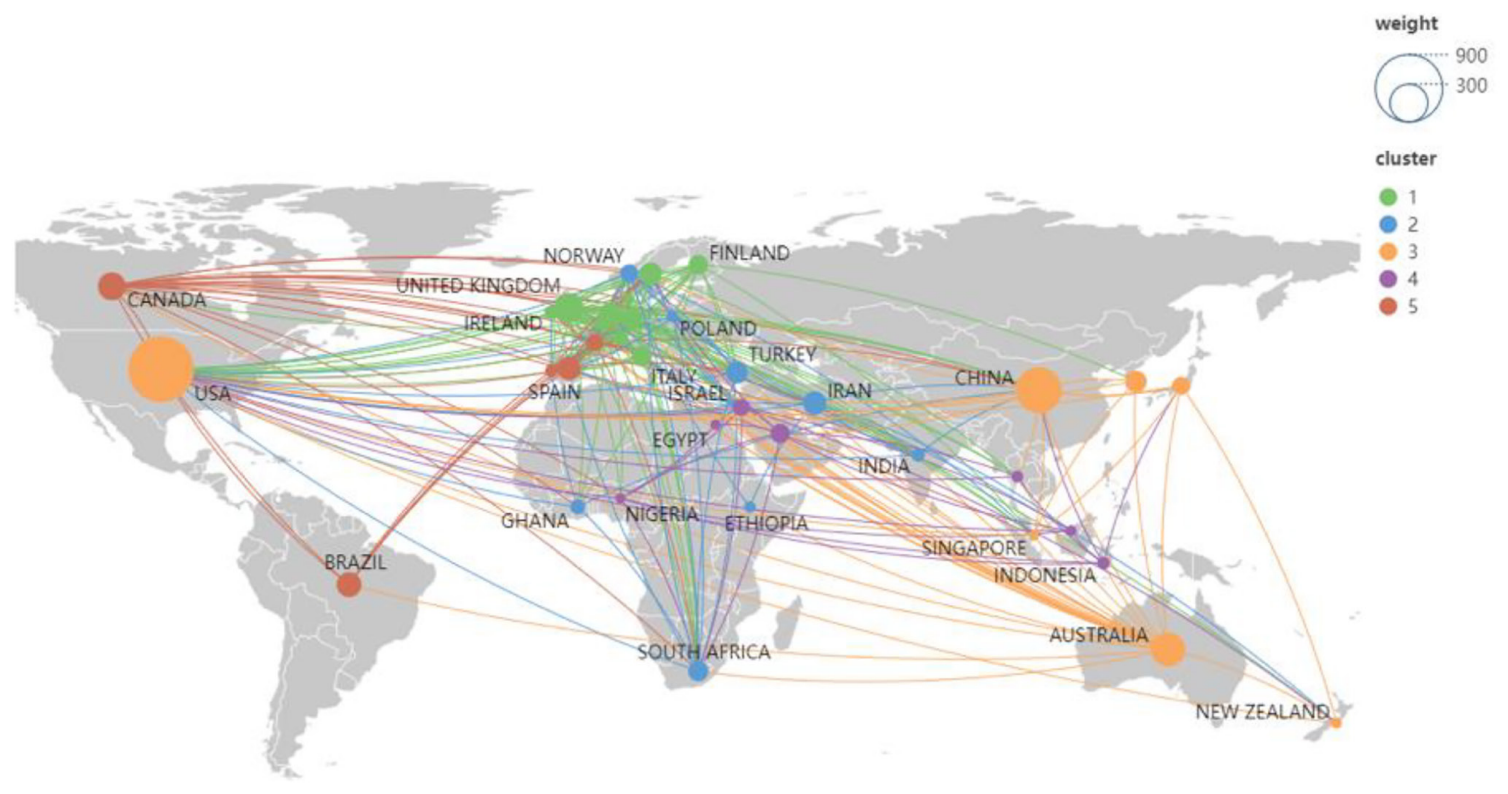
Network visualization analysis of countries/regions.

A total of 42 countries (regions) published papers on nursing management, with relatively concentrated distributions, mainly in North America, China, Europe, and Australia. The United States had the highest number of papers, with 791 (25.77%), far greater than that of any other country. China (386), Australia (212), England (162), Canada (144), and Brazil (114) had approximately 12.58%, 6.91%, 5.28%, 4.69%, and 3.71% of the published papers, respectively. Figure 3 shows the country/region collaboration network created via the coauthor analysis method. We used the size of the nodes to represent the number of papers published by each country and the links between nodes to represent collaboration. The thickness of the connection reflects the strength of the cooperation. In terms of connection strength, the cooperative ties among North America, Europe, and Australia are relatively close. China should further enhance research cooperation with these regions.

### 3.3. Analysis of institutions/universities

**Figure 4.**
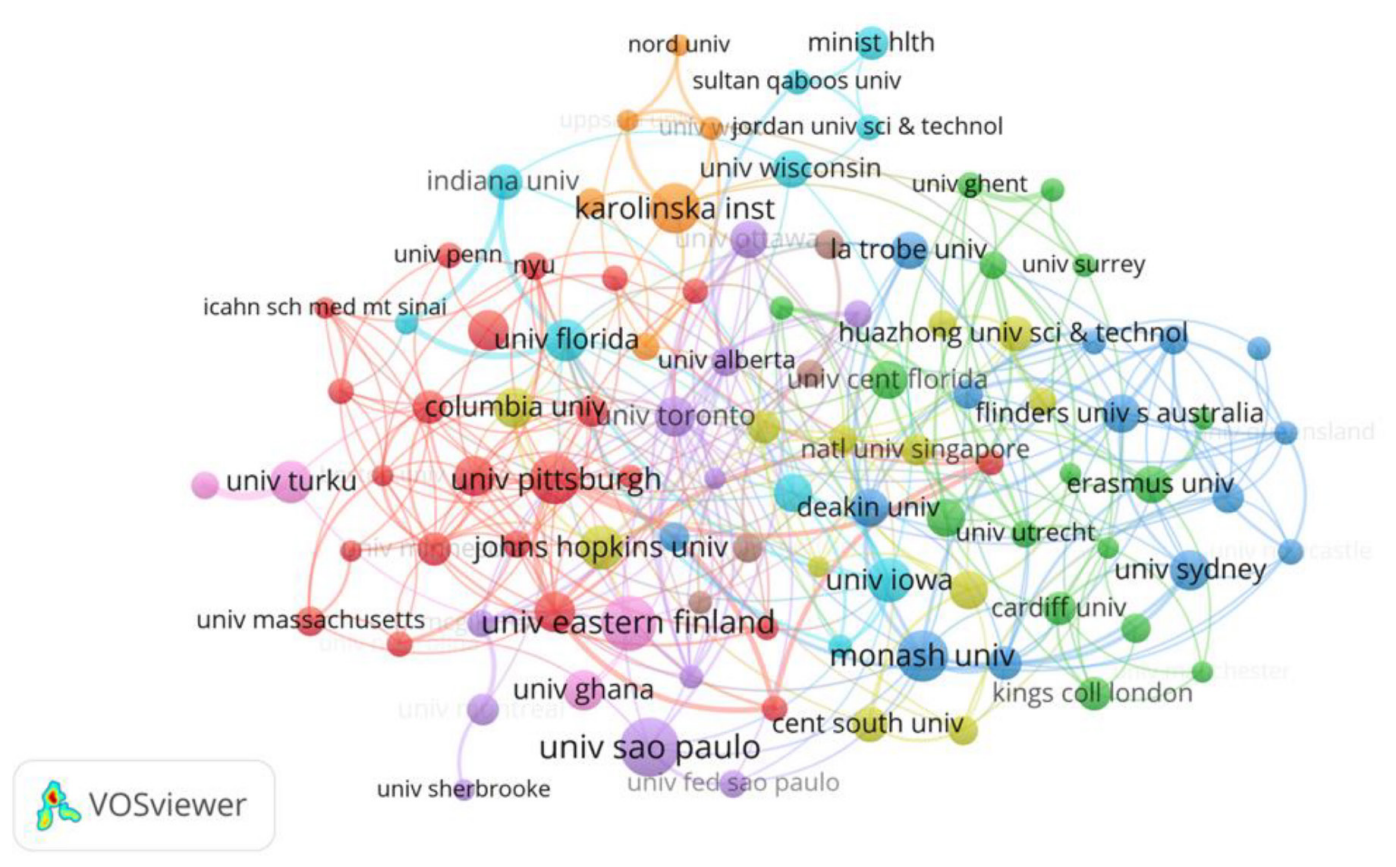
Network visualization analysis of research institutions/universities.

A total of 99 research institutions/universities published papers on nursing management, with a relatively dispersed distribution. The University of São Paulo in Brazil published the highest number of papers, with 30 (0.98%). The remaining institutions with more than 20 published papers were the University of Eastern Finland (28, 0.91%), Monash University (26, 0.85%), Karolinska Institutet (25, 0.81%), and the University of Pittsburgh (25, 0.81%). A research institution collaboration network, as illustrated in Figure 4, was created via the coauthor analysis method. The larger the nodes in the graph are, the more papers were published by the research institutions/universities. A thicker line and a shorter distance between nodes indicate stronger cooperation between research institutions/universities. Different colors represent different clusters. These institutions/universities represent the main academic groups in global nursing management research at present. Scholars engaged in this field should focus on strengthening communication with them.

### 3.4. Analysis of journals

**Figure 5.**
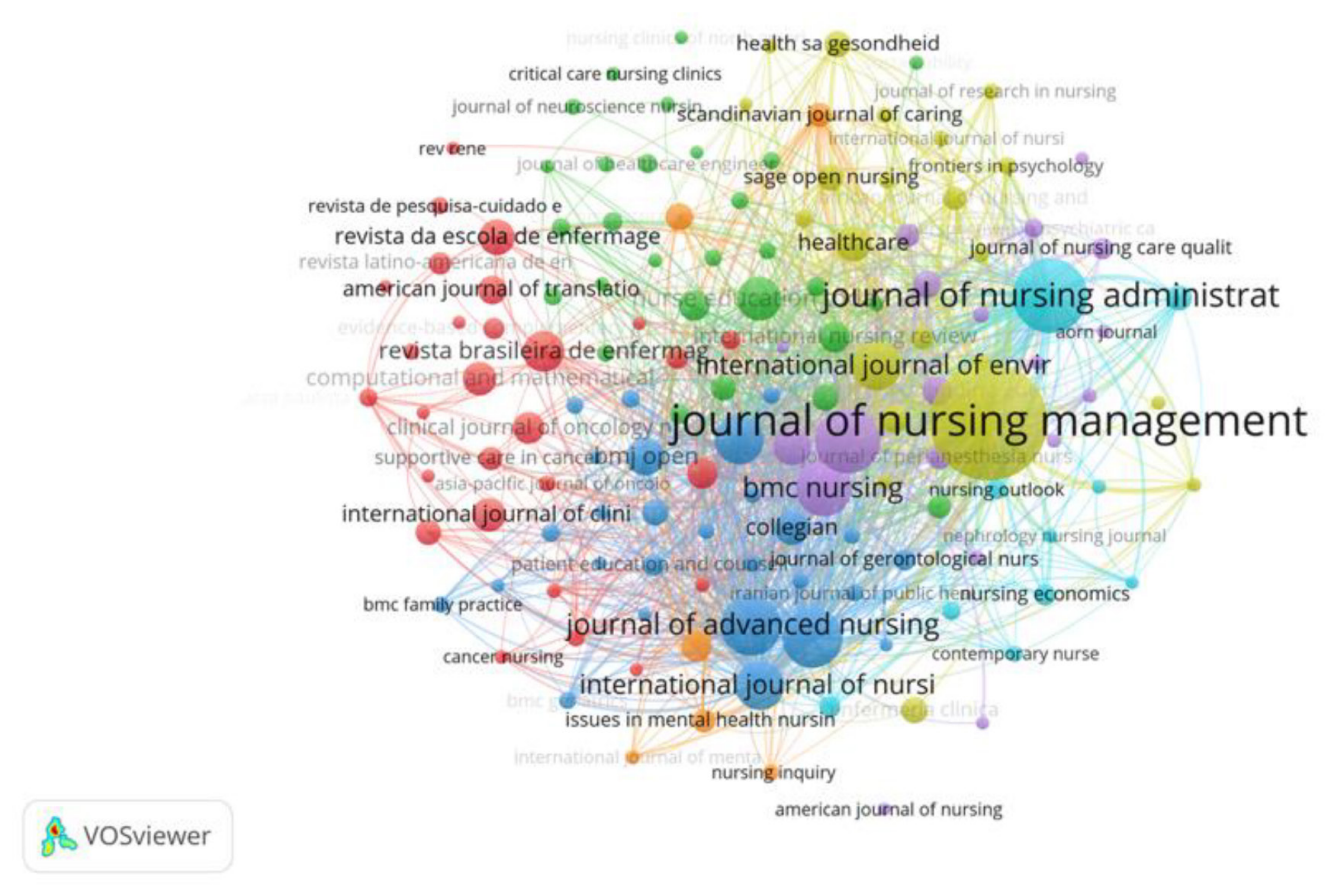
Citation network of journals in nursing management.

In the past 10 years, 140 journals have published papers on nursing management. Among them, the journal with the greatest number of papers was the *Journal of Nursing Management* (IF = 4.4), with 187, followed by the *Journal of Nursing Administration* and *Pain Management Nursing*, with IFs of 1.9 and 2.5, respectively. The *Journal of Nursing Management* is the most authoritative journal for supporting and illuminating the practice of nursing and health care management, innovation, and leadership. The top 3 journals in terms of the number of published papers are mainly nursing management research journals published by European and American countries. The citation network of journals in nursing management is shown in Figure 5.

### 3.5. Analysis of authors

**Figure 6.**
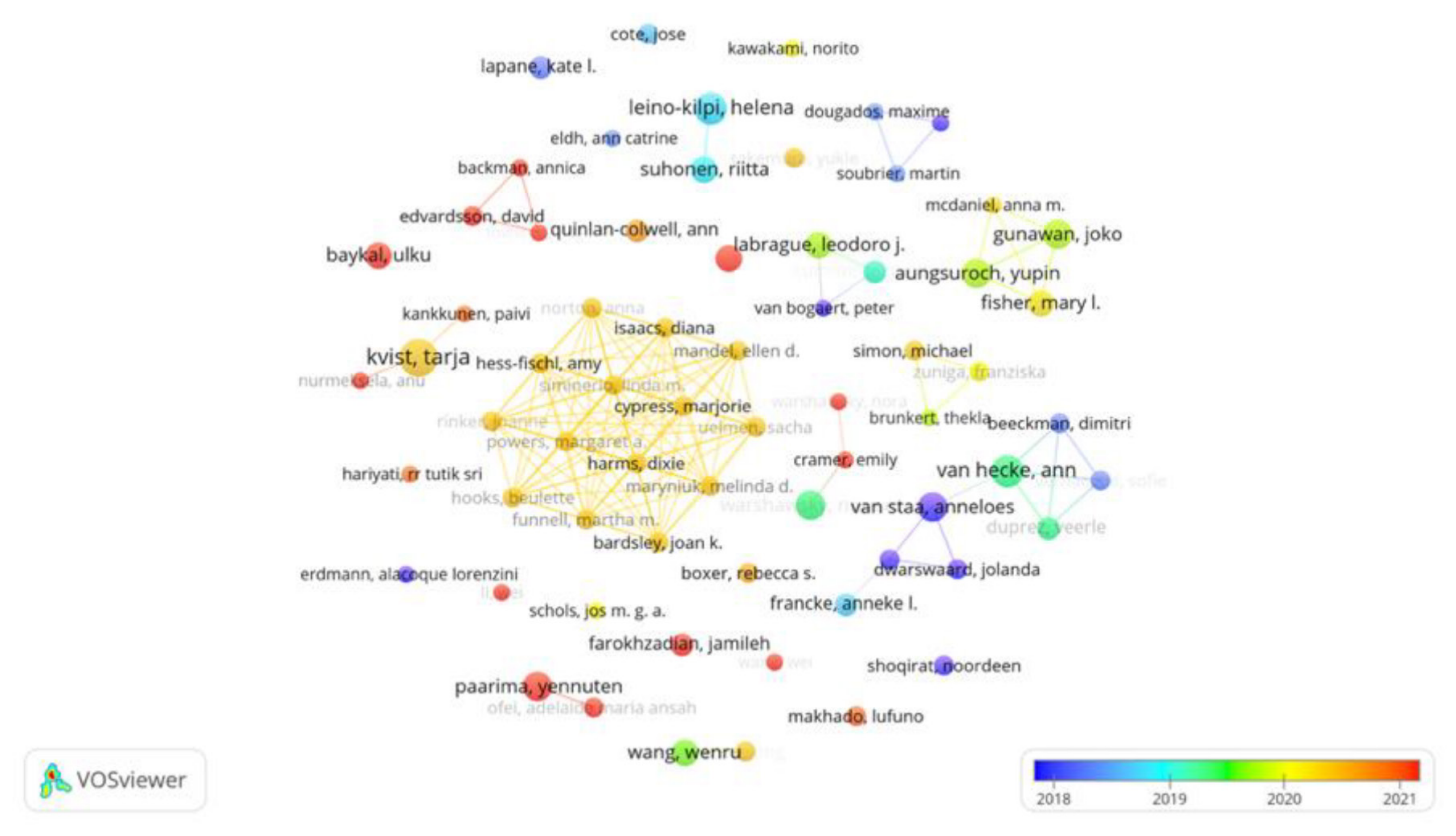
Citation network of cooperative research of the 67 authors.

A total of 67 authors published papers related to nursing management, and Table 1 lists the top 10 authors by publication count. A total of 93 papers were published by the top 10 authors, accounting for 3.03% of all the literature in the field. The most productive author of research articles on nursing management was Tarja Kvist. Among the top 10 authors, Tarja Kvist is from Finland, and the remaining 2 are from Belgium and Finland. Our study revealed that although Tarja Kvist published the most articles on nursing management, Helena Leino-Kilpi had the highest H-index, indicating that she had the greatest influence in this research field. Tarja Kvist is a Finnish nursing expert, mainly employed in the Department of Nursing Sciences at the University of Eastern Finland. Her research mainly focuses on the quality of clinical care, patient safety, and health management for specific populations. Helena Leino-Kilpi is a professor in the Department of Nursing Sciences at the University of Turku in Finland. She previously served as the dean of the School of Nursing at the University of Turku and has long been engaged in research in the fields of nursing ethics, patient empowerment, perioperative nursing quality, and nursing education. It is evident that European scholars led by those from Finland play an important role in this research field. An analysis of the cooperative research of the 67 authors is shown in Figure 6. The top 10 authors by number of publications are shown in Table 1. Most authors shared connections, indicating the establishment of cooperative relationships, with a small number of non-collaborators around the cooperative network.

**Table 1:** The top 10 authors by number of publications.

Rank	Author	H-index	Total citations	N	Country
1	Tarja Kvist	22	158	12	Finland
2	Ann Van Hecke	32	133	10	Belgium
3	Helena Leino-Kilpi	50	164	10	Finland
4	Yupin Aungsuroch	16	139	9	Thailand
5	Joko Gunawan	14	139	9	Thailand
6	Yennuten Paarima	4	47	9	Ghana
7	AnneLoes van Staa	24	240	9	Netherlands
8	Nora E. Warshawsky	15	165	9	United States
9	Ulku Baykal	9	23	8	Turkey
10	Mary L. Fisher	11	94	8	United States

### 3.6. Analysis of burst keywords and co-occurrence keywords

**Figure 7.**
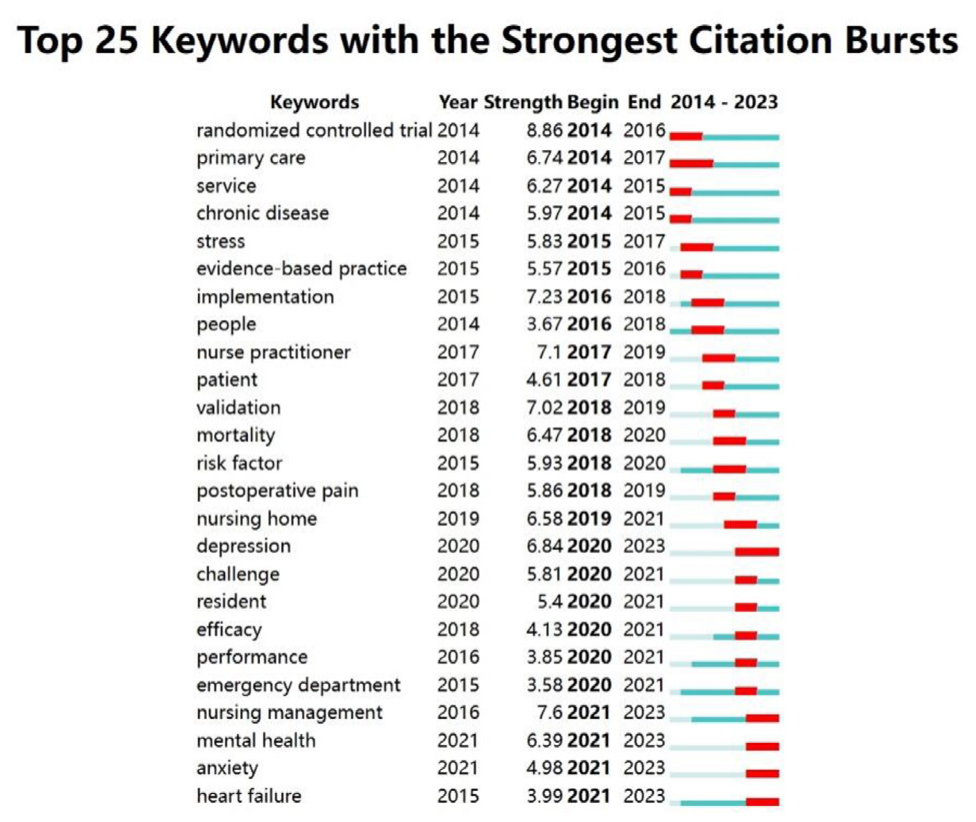
Top 25 keywords with the strongest citation bursts in nursing management.

**Figure 8.**
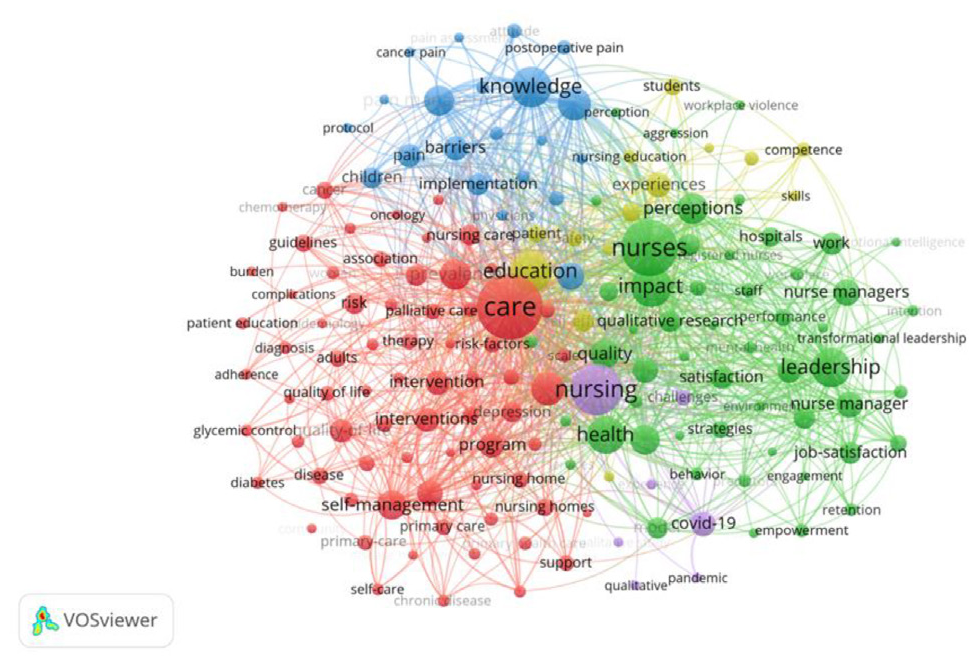
The co-occurrence of the keyword in nursing management. The co-occurrence of the keyword in the field. All keywords were divided into 3 clusters and colored differently: pain nursing management (in blue), nursing quality management (in green), and disease nursing management (in red).

**Figure 9.**
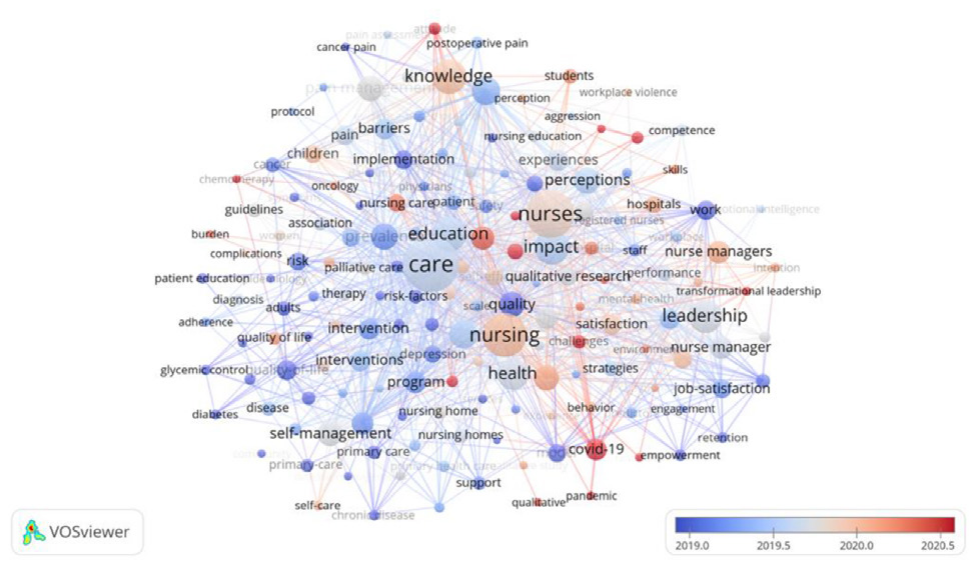
Distribution of keywords based on the average time of appearance. Red represents a recent appearance, and blue represents an early appearance. The line between 2 keywords represents their co-occurrence in the same publication, with a thicker line indicating a closer relationship.

Keywords are professional terms that can summarize the basic content of a research article. We used CiteSpace to generate the top 25 keywords with the strongest citation bursts, and Figure 7 shows the generated keyword burst graph. Among the top 25 keywords, those with the strongest bursts were randomized controlled trial, nursing management, and implementation. The keywords with the longest duration of burst were depression and primary care. Among these keywords, depression, nursing management, mental health, anxiety, and heart failure became more prevalent in recent years. Figure 8 visualizes the keyword co-occurrence analysis. All keywords were divided into 3 major clusters and colored differently: pain nursing management (in blue), nursing quality management (in green), and disease nursing management (in red). In addition, there are smaller research clusters such as nursing education management (in yellow) and COVID-19 nursing management (in purple). These clusters are currently the main branches of nursing management research. Figure 9 shows the distributions of keywords based on the average time of appearance. Red represents a recent appearance, and blue represents an early appearance. The line between 2 keywords represents their co-occurrence in the same publication, with a thicker line indicating a closer relationship. It is evident that nursing education management and COVID-19 nursing management are new research areas that have emerged in recent years.

### 3.7. Cluster analysis of keywords

**Figure 10.**
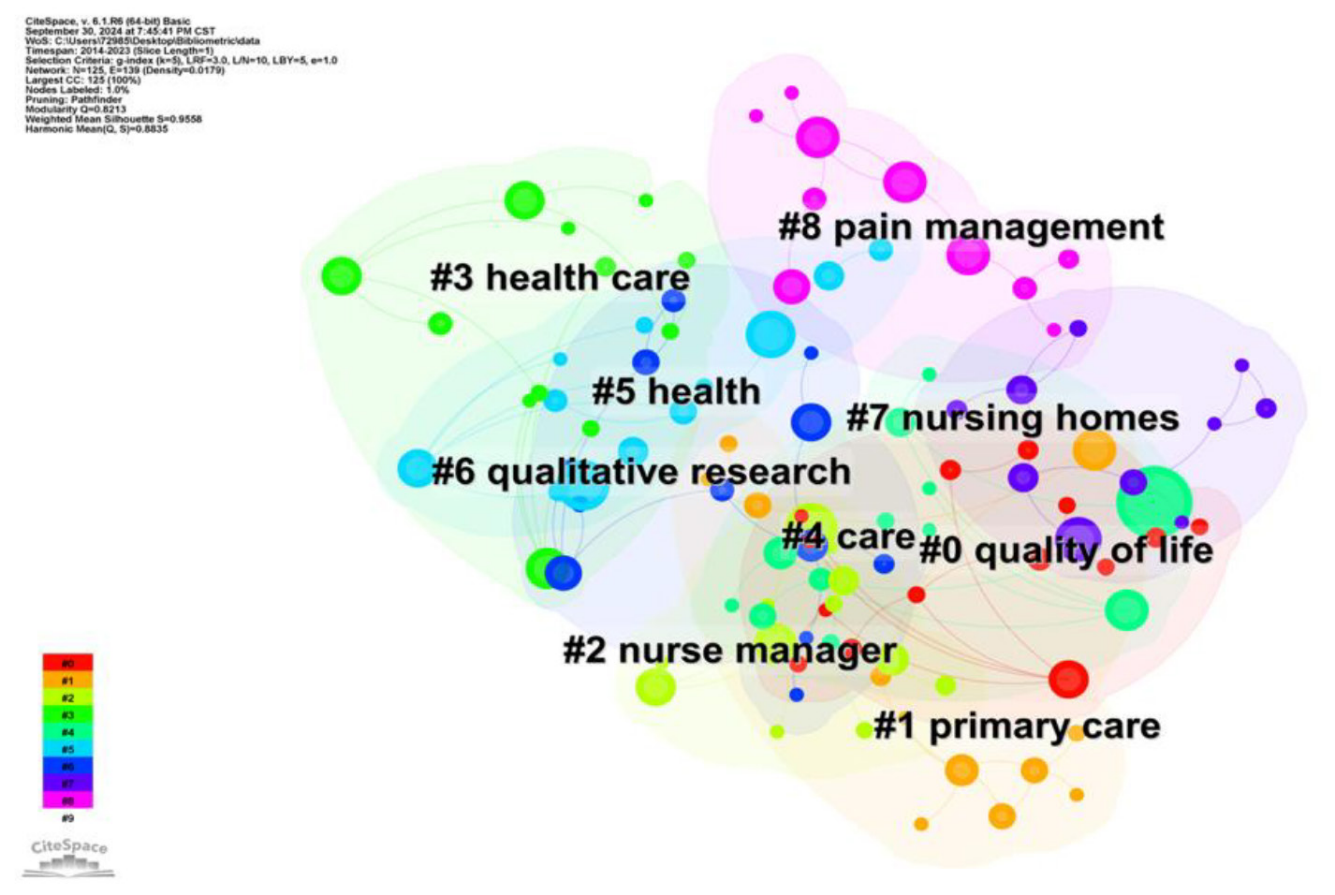
Cluster analysis of keywords in nursing management.

Keyword clustering classifies similar keywords to reveal research topics and hotspots and to form a clear research context. We conducted cluster analysis on the high-frequency keywords of the 3069 papers. The 9 largest clusters of keywords produced by the log-likelihood ratio included quality of life, primary care, nurse manager, health care, care, health, qualitative research, nursing homes, and pain management (Table 2). Figure 10 shows the 9 largest clusters of keywords.

**Table 2:** Research hotspots in the clustering of high-frequency keywords in nursing management in the WoSCC from 2014 to 2023.

Clustering	Research hotspot	Keywords
0	Quality of life	palliative care, nurisng homes, symptom management, physical restraint, advance care planning
1	Primary care	chronic disease, case management, patient outcome assessment, chronic obstructive pulmonary disease, nurse-led clinics
2	Nurse manager	nurse performance behavior, cultural diversity, shift, nurse unit manager
3	Health care	health care, Africa, organizational communication, health services management, health professionals
4	Care	Nursing care, infection prevention, intravenous catheterization, professional education, actor-network theory
5	Health	Job satisfaction, nursing managers, health workforce, primary health care, nurse-head nurse trust
6	Qualitative research	Self-management support, palliative care, disease management, chronic obstructive pulmonary disease, behavior change support
7	Nursing homes	Evidence-based practice, mixed methods, evidence-based practice beliefs, evidence-based practice behavious, cancer pain management
8	Pain management	Quality improvement, inquiry-based learning, nutrition management, interprofessional collaboration, interprofessional education

## 4. Discussion

### 4.1. International research on nursing management has been active

Due to highly standardized and diversified nursing needs, the general nursing management model has struggled to meet the nursing needs of patients.^[8,9]^ The nursing refinement process is based on routine nursing work, and it is necessary to follow scientific procedural principles to further refine nursing management practice.^[10]^ Therefore, nursing management research has attracted increasing attention from various countries. Over the past decade, many countries in North America, Asia, Europe, and Oceania have participated in nursing management research. Through bibliometric analysis, it was found that regional cooperation has been continuously strengthened. In terms of the number of published papers, the United States leads in nursing management research, followed by China. Nursing management research in the 2 countries was relatively active for many common reasons, but also due to many differences.^[11]^ We analyzed the possible causes of this difference by reviewing the literature. First, both countries have some of the largest medical systems in the world. Second, there are a large number of nursing practitioners in both countries, and the nursing human resources situation is complex. Third, both countries have developed economies and societies with high demands for the quality and safety of nursing. Nursing management research in the United States is influenced by the market and medical insurance policies. Meanwhile, there is a relative shortage of nursing staff, which creates a higher demand for effective nursing management. However, the United States has a long history of research and benefits from a mature academic research environment and research funding. In China, research in this area is mainly influenced by national policies. The huge population has given rise to increased demand for nursing management. Meanwhile, the scale of medical institutions in China has expanded rapidly, and the requirements for the quality of nursing management have gradually increased.^[12]^

There is a clear trend of growth in the number of nursing management papers, with the number of papers published in 2023 more than tripling compared to 2014. The quality of the papers has also improved significantly. We speculate that this growth is related to many factors, such as the development of many emerging research institutions, the emergence of COVID-19 nursing management, and the increasing demand for nursing education management. Although the bibliometric results did not clearly reflect the reasons for this change, through the analysis of the literature, we believe that the development of AI, population aging, improvements in the quality of life, and the demand for primary health care may also have promoted the development of nursing management research.^[13]^ Due to close international cooperation and exchanges, as well as the increased cross-border mobility of nursing staff, regions with higher levels of nursing management, such as the United States, China, and Europe, may attract more high-quality nursing personnel, take the leading position in international cooperation and nursing leadership, and have greater influence. In regions with a relatively high level of nursing management, it is often easier to apply digital health and AI in nursing work.

### 4.2. The trend of centralized cooperation in nursing management research is becoming more and more obvious

With increasingly frequent academic exchanges, cooperation has become mainstream in academic research. In the past decade, the trend of cooperation in the field of nursing management research has become increasingly evident.^[4]^ Through a bibliometric analysis of countries, institutions, and authors in nursing management papers over the past decade, this study revealed that highly productive authors tend to be more collaborative and that exchanges between academic institutions in developed countries are more common. Collaborative research in this area has produced many high-quality research papers and multicenter research results, which provide support for the development of nursing science. Meanwhile, in the past decade, the international cooperation models and scope in global nursing management research have also undergone changes. We analyzed and believe that this might be related to many deep-seated reasons. First of all, countries around the world are generally confronted with problems such as nurse shortages, attrition, and job burnout, and the shortage of nursing human resources has become a global issue.^[14]^ Second, research cooperation in nursing management among low- and middle-income countries has been strengthened, and the diversification and globalization of nursing knowledge have developed. Third, global public health issues such as COVID-19 and climate change have made it necessary for health institutions around the world to enhance cooperation and promote the strengthening of cross-border collaboration networks in nursing management.^[15]^

The research focus of nursing management has continuously evolved over time. It has shifted from emphasizing primary care, chronic disease management, and the improvement of nursing service quality to addressing mental health, nursing education, and patients’ quality of life. Moreover, the scope of research has progressed from clinical disease management toward addressing deep-seated problems and innovating management methods. The advancement of digital health has transformed nursing management practices while also driving a surge in the number of studies and multinational collaborative research in recent years. Over time, the educational requirements for nursing practice have risen, leading nursing management to place greater emphasis on education-related management. Research on COVID-19-related nursing management experienced explosive growth over a short period, but as the epidemic has waned, the popularity of related research has declined.

### 4.3. Nursing management pays more attention to quality of life, psychological state, and primary health care

Since the beginning of the 21st century, with economic and social development as well as population aging in the world’s major developed countries, the pace of people’s lives has accelerated; the proportion of elderly individuals in the population has increased; new demands such as psychological nursing intervention, public health care, and elderly care have increased; and the research focus of nursing management has also changed.^[16,17]^ In terms of research content, research directions have shifted from nursing management for some specific diseases and specific groups to nursing management for quality of life, psychological status, and primary health care, with a special emphasis on attending to the essence of nursing, improving the quality of patient care, and enhancing the overall nursing level of society.^[18]^ The transformation of research content in nursing management was due to the fundamental change in the disease spectrum and health concepts. The connotation of health has expanded. Psychological conditions and quality of life are regarded as important aspects of health and have also received attention in nursing management research.^[19]^ Primary care is the cornerstone of health. Due to increased investment in primary care by various countries in recent years, the requirements for the level of nursing management in primary care have also risen accordingly.

### 4.4. The potential implications of the findings for future nursing management research and practice

Based on the results of this bibliometric study and in combination with the latest research literature on nursing management, we made reasonable inferences about future research trends. In future research on nursing management, we predict that the United States, China and Europe will still hold a dominant position. Although the number of articles published in China has grown rapidly over the past decade, the country still needs to enhance cooperation and exchanges with Europe and the United States. The institutions/universities with the largest number of published papers, the most active authors, and the most important journals are mainly concentrated in Europe. This indicates that the European region has a profound foundation in nursing management research and may still influence the research direction in this field in the future. Over the past decade, many changes have also been observed in the characteristics of global nursing research. Research cooperation on nursing management among low- and middle-income countries has been strengthened, and they have formulated corresponding research strategies based on their own contexts. Healthcare policies have had a significant impact on the research direction of nursing management, and primary health care has received increased attention.^[20]^ The research and development of AI in nursing management have a relatively short history and have not accumulated sufficient literature, but this research trend has attracted our attention. Although COVID-19-related nursing management has only been intensively studied for a relatively short period of time, it has objectively strengthened the construction of the global network for nursing management of public health issues, enhanced the research capacity for nursing management of epidemic diseases, and indirectly promoted the improvement of nursing management levels in low- and middle-income countries.

## 5. Limitations

Our study has several unavoidable limitations. First, for the convenience of bibliometric research, we only included English literature, which might overlook potentially valuable studies in other languages. Including only English literature may introduce potential biases in the research results, as research articles published in countries where English is not the main language may be underrepresented. Second, we only relied on the WoSCC database for screening, thus excluding other important databases. This increases the possibility of missing relevant papers from these databases. Although the scope of the WoSCC database was considered comprehensive, it does not fully cover all academic literature. Therefore, the findings were limited to the WoSCC and English-language publications. Third, the bibliometric software tools used have inherent methodological limitations. The mechanical nature of the CiteSpace and VOSviewer tools employed in this paper may have led to the imprecise extraction of keywords and incomplete analysis of article content. Single research evaluations through bibliometrics are not comprehensive, and citation counts can be influenced by factors such as outdated research and publication dates. Fourth, by excluding conference literature, there may be citation bias. Many newly published studies have not accumulated many citations yet, which may affect the results. Despite meticulous attempts to read and analyze all articles encompassed in this study, the possibility of residual researcher bias cannot be completely ruled out. By searching relevant literature, we found that the search terms we selected could cover the main contents of nursing management, but there was a possibility of missing related alternative terminology (such as nursing leadership, workforce management, staffing, governance), resulting in an incomplete retrieval of papers.

## 6. Conclusion

In summary, this study used bibliometrics to analyze English papers on nursing management that were published over a 10-year period from 2014 to 2023. The trend of growth in nursing management research was explored based on the number of annual publications, and authoritative academic journals related to nursing management were identified based on the number of published articles. In addition, the academic leaders and main research institutions that contributed to this research direction were determined by analyzing the authors and institutions. Moreover, based on keyword clustering and topic evolution analysis, the popular research topics and development trends of nursing management were discussed. Our study revealed that with economic and social development and demographic changes, nursing management has paid increasing attention to patients’ quality of life, psychological state, and primary health care in the past 10 years. Therefore, the findings of this study will be very helpful in understanding future research directions for nursing management and will positively guide the formulation of nursing management policies and the development of the nursing profession.

## Acknowledgments

We thank those who participated in and supported this study.

## Author contributions

**Conceptualization:** Yining Wang, Hailin Chu, Ya Xin, Xuyao Liu, Yongxia Ren.

**Data curation:** Yining Wang, Hailin Chu, Ya Xin, Yongxia Ren.

**Formal analysis:** Yining Wang, Hailin Chu, Ya Xin.

**Funding acquisition:** Yining Wang, Hailin Chu, Yongxia Ren.

**Investigation:** Yining Wang.

**Methodology:** Yining Wang, Xuyao Liu.

**Project administration:** Yining Wang, Xuyao Liu.

**Resources:** Yining Wang.

**Software:** Yining Wang, Hailin Chu, Xuyao Liu.

**Supervision:** Xuyao Liu.

**Validation:** Xuyao Liu.

**Writing – original draft:** Yining Wang, Xuyao Liu.

**Writing – review & editing:** Yining Wang, Hailin Chu, Ya Xin, Yongxia Ren.

## References

[1] Li XR Luo QL. Effects of high-quality neurosurgical nursing care on improving clinical nursing quality. World J Clin Cases. 2024;12:4999–5007. 39109026 10.12998/wjcc.v12.i22.4999 PMC11238798

[2] Galiano MA Moreno Fergusson ME Guerrero WJ. Technological innovation for workload allocation in nursing care management: an integrative review. F1000Res. 2024;12:104. 38434658 10.12688/f1000research.125421.3 PMC10904957

[3] Yang J Mao T Yuan P Zhou J Li M Chen B. The influence of the personality traits of newly graduated nurses on the knowledge, skills and professional self-efficacy in standardized training: a cross-sectional study. BMC Nurs. 2024;23:731. 39379896 10.1186/s12912-024-02392-z PMC11463070

[4] Moraca E Zaghini F Fiorini J Sili A. Nursing leadership style and error management culture: a scoping review. Leadership Health Services. 2024;37:526–47. 10.1108/LHS-12-2023-0099 39344575

[5] Cachata D Costa M Magalhaes T Gaspar F. The integration of information technology in the management and organization of nursing care in a hospital environment: a scoping review. Int J Environ Res Public Health. 2024;21:968. 39200579 10.3390/ijerph21080968 PMC11353944

[6] Laine H Heikkilä A Peltonen LM Smed J. Digital information management for advanced practice nursing: needs assessment. Stud Health Technol Inform. 2023;302:617–8. 37203764 10.3233/SHTI230221

[7] Hassan W Duarte AE. Bibliometric analysis: a few suggestions. Curr Probl Cardiol. 2024;49:102640. 38740289 10.1016/j.cpcardiol.2024.102640

[8] Chang CY Jen HJ Su WS. Trends in artificial intelligence in nursing: impacts on nursing management. J Nurs Manag. 2022;30:3644–53. 35970485 10.1111/jonm.13770

[9] Su WS Hwang GJ Chang CY. Bibliometric analysis of core competencies associated nursing management publications. J Nurs Manag. 2022;30:2869–80. 36076321 10.1111/jonm.13795

[10] Chu Y Kong Y Zhang J Zhang B Song J. Effect of fine process management on nursing management efficiency and patient satisfaction in gastric cancer operating room. Panminerva Med. 2024;66:88–90. 34609122 10.23736/S0031-0808.21.04569-9

[11] Zhou L Godsey JA Kallmeyer R Hayes T Cai EL. Public perceptions of the brand image of nursing: cross-cultural differences between the United States and China. Nurs Outlook. 2024;72:102220. 38878616 10.1016/j.outlook.2024.102220

[12] Xie J Liu M Ding S. Time management disposition and related factors among nursing managers in China: a cross-sectional study. J Nurs Manag. 2020;28:63–71. 31644829 10.1111/jonm.12890

[13] Gonzalez-Garcia A Pérez-González S Benavides C Pinto-Carral A Quiroga-Sánchez E Marqués-Sánchez P. Impact of artificial intelligence-based technology on nurse management: a systematic review. J Nurs Manag. 2024;2024:3537964. 40224848 10.1155/2024/3537964 PMC11919197

[14] Michaeli DT Michaeli JC Albers S Michaeli T. The healthcare workforce shortage of nurses and physicians: practice, theory, evidence, and ways forward. Policy Polit Nurs Pract. 2024;25:216–27. 39396540 10.1177/15271544241286083

[15] Farahani MA Nargesi S Saniee N Dolatshahi Z Heidari Beni F Shariatpanahi S. Factors affecting nurses retention during the COVID-19 pandemic: a systematic review. Hum Resour Health. 2024;22:78. 39567984 10.1186/s12960-024-00960-7 PMC11580645

[16] Wang J Anderson R Perez JS. Understanding adaptive leadership in the context of nursing homes. J Appl Gerontol. 2024;43:1524–35. 38566520 10.1177/07334648241243312

[17] Vara-Ortiz MA Marcos-Alonso S Fabrellas-Padrés N. Experience of primary care nurses applying nurse-led management of patients with acute minor illnesses. Int J Nurs Pract. 2024;30:e13216. 37964496 10.1111/ijn.13216

[18] Meyer C Hickson L. Nursing management of hearing impairment in nursing facility residents. J Gerontol Nurs. 2020;46:15–25. 10.3928/00989134-20200605-04 32597997

[19] Liu Q Li Z. Multidisciplinary collaborative nursing management improves health behaviors and psychological status in patients with diabetes mellitus and coronary heart disease. J Multidiscip Healthc. 2026;19:574565. 41867420 10.2147/JMDH.S574565 PMC13003807

[20] Ortiz MR. Health and nursing policies: word choice and crafting policies with unique nursing knowledge. Nurs Sci Q. 2023;36:85–8. 36571312 10.1177/08943184221131961

